# Spatial distribution and prognostic value of tumor-associated macrophages in head and neck squamous cell carcinomas

**DOI:** 10.3389/fonc.2026.1806162

**Published:** 2026-05-08

**Authors:** Miray-Su Yılmaz Topçuoğlu, David Krum, Rolf Warta, Catharina Lotsch, Oktay Kütük, Carolin Mogler, Niels Grabe, Patrick J. Schuler, Gerhard Dyckhoff, Christel Herold-Mende

**Affiliations:** 1 Department of Otorhinolaryngology, Head and Neck Surgery, University of Heidelberg, Medical Faculty of Heidelberg, Heidelberg, Germany; 2 Department of Otorhinolaryngology, Head and Neck Surgery, Klinikum Kassel, Kassel, Germany; 3 Division of Experimental Neurosurgery, Department of Neurosurgery, University of Heidelberg, Medical Faculty of Heidelberg, Heidelberg, Germany; 4 Institute of Pathology, TUM School of Medicine, Technical University of Munich, Munich, Germany; 5 Hamamatsu Tissue Imaging and Analysis Center, BioQuant, University of Heidelberg, Heidelberg, Germany

**Keywords:** HNSCC, p16-status, survival, tumor cell nest, tumor stroma, tumor-associated macrophages

## Abstract

**Introduction:**

Tumor-associated macrophages (TAMs) belong to the most frequent immune cells in the tumor microenvironment of head and neck squamous cell carcinomas (HNSCC). They can undergo an anti- or pro-tumoral polarization, the latter often referred to as M2-like activation. Because existing data are either not consistent, not well-connected to clinicopathological parameters or lack a detailed spatial distribution, we aimed to get more insight into the relevance of this immune cell population and their activation status.

**Methods:**

This study analyzed the spatial distribution and prognostic value of CD68+TAMs and CD68+CD163+M2-like TAMs in different tumor compartments and tumor-distant stromal areas, in 85 treatment-naïve HNSCC. Various clinicopathological features were considered, such as major tumor sites, stage, T-stage, nodal status and p16-status. TAM and M2-like TAM densities were analyzed using multicolor immunofluorescence stainings followed by an objective tissue cytometry-based quantification at the single-cell level in whole tissue sections and subsequent uni- and multivariate survival analyses.

**Results:**

Whereas we observed higher TAM and M2-like TAM densities in p16-negative HNSCC specimens, densities of M2-like TAMs were highest in the tumor-near stroma of advanced nodal-positive p16-negative HNSCC compared to tumor cell nests and tumor-distant stroma, particularly in younger and male patients and patients with hypopharynx carcinomas. Moreover, higher infiltration of M2-like TAMs turned out to be an independent prognostic factor of poorer survival even exceeding the impact of the p16-status and the tumor site.

**Discussion:**

In summary, our data provide a strong rationale to target M2-like TAMs to improve success of immune-modulatory treatments and survival of patients suffering from p16-negative HNSCC.

## 1 Introduction

Head and neck cancers present one of the most common types of tumors worldwide (1–3), and currently there is growing incidence of affected younger patients. This is largely due to an increase of human papilloma virus (HPV)-driven head and neck squamous cell carcinomas (HNSCC) (4, 5). Histologically, HNSCCs are the most prevalent head and neck tumors (6, 7). Depending on stage, T-stage, detection of lymph node and distant metastases, p16-status, and tumor recurrences, the 5-year survival rate ranges between 30% and 80% (8–10). Despite intensive research efforts, standard treatment modalities for HNSCC patients are still limited to surgery, radiation, and platin-based chemotherapy, as well as to selected antibody- or immunotherapies (10–12). The p16-status, which serves as surrogate parameter for HPV-driven HNSCC, is routinely assessed, because its identification has implications for treatment strategies and is associated with an improved prognosis. Therefore, HPV-positive carcinomas are regarded as distinct tumor entities (13). For instance, HPV-positive carcinomas often respond better to therapeutic regimens due to their distinct tumor- and immunobiology, opening novel therapeutic options for this tumor entity, in particular regarding T cell-based immunotherapies (14, 15). However, despite improvements in the classification and treatment options of HNSCC, survival rates remain low and have not significantly improved in recent years (16). Consequently, further therapeutic approaches are urgently needed to achieve better treatment outcomes and improved survival rates. Among them, targeting immunosuppressive tumor-associated macrophages (TAMs) has emerged as a promising strategy (17–19). TAMs belong to the most prevalent immune cell types in the tumor microenvironment (TME) of various carcinomas, residing in tumor cell nests as well as in the tumor-adjacent stroma (19–22), which has also been reported for HNSCC (21, 23). Furthermore, a higher number of TAMs, particularly of anti-inflammatory and pro-tumoral activated TAMs, is associated with a poor prognosis in several types of solid cancer (20, 24–26), which has been demonstrated among others in lung, pancreatic, prostate and breast cancer (22, 27–29). In HNSCC, a pro-tumoral activation of TAMs might also play a role in the therapeutic resistance to anticancer drugs (20). Depending on their activation, macrophages can have different phenotypes and functions in the TME. Pro-inflammatory, so-called M1-like polarized TAMs can act as anti-tumoral immune cells by promoting phagocytosis and antigen presentation, while M2-like polarized, anti-inflammatory TAMs play a pro-tumoral role and are characterized by the secretion of tumor-growth-promoting and immunosuppressive cyto- and chemokines (17, 20, 26). High expression of scavenger receptors (CD163, CD204 and CD206), immune checkpoint ligands (PD-L1) and anti-inflammatory cytokine secretion (IL-10, TGF-ß) occur predominantly in M2-like TAMs (18, 26, 30, 31).

Some previous studies have investigated the role of TAMs in HNSCC, but none of them specifically focused on pro-tumoral M2-like TAMs (32, 33). Other studies performed investigations on M2-like TAMs and their prognostic role, but have largely focused on cancers of the oral cavity and only rarely considered other HNSCC sites such as the oropharynx, larynx, and hypopharynx (16, 21, 32, 34). Furthermore, the association of immunosuppressive TAMs and HPV status in HNSCC is still not fully understood and demands further investigations (21, 24, 30, 35). Therefore, this study analyzed the distribution of pro-tumoral M2-like TAMs in a large, clinically well-annotated cohort of 85 HNSCC specimens using tissue cytometry. Notably, our analysis covered various tumor sites, including the oral cavity, oropharynx, larynx, and hypopharynx and we assessed M2-like TAM densities in association with other clinicopathological parameters. Moreover, this study analyzed in more detail the spatial distribution of M2-like TAMs within HNSCC tumor specimens, particularly regarding tumor cell nests and surrounding tumor stroma, as well as the relationship between M2-like TAMs and p16-status in HNSCC.

## 2 Materials and methods

### 2.1 Patients and tumor samples

A total of 85 treatment-naïve HNSCC specimens were intraoperatively obtained from patients treated at the Department of Otorhinolaryngology, Head and Neck Surgery at the University Hospital Heidelberg, Germany. Clinical parameters of 84 of the specimens have been described previously (36). The use of patient material was approved by the ethics committee at the University Hospital of Heidelberg (S-70/99, amendment 09/01/2004). Written informed consent was obtained from all patients involved in the study. Tumors were staged according to the Union for International Cancer Control (UICC) TNM classification, following the 7^th^ edition used at the time of diagnosis. None of the patients had distant metastases at the time of diagnosis. Patient characteristics are summarized in **Table 1**. Patient characteristics for the 77 p16-negative specimens are summarized in **Supplementary Table 1**. Tissue samples were immediately snap frozen after surgery and stored at -80 °C until further processing. For subsequent analysis, we only included cases with a tumor cell content of at least 60%, as confirmed by an experienced pathologist (C.M.) for all samples. We focused specifically on immunosuppressive M2-like TAMs, as our methodological approach enables their robust and reproducible identification. Although TAMs are known to be a heterogeneous population, comprehensive characterization of all TAM subpopulations was beyond the scope of this study.

**Table 1:** Patient characteristics of 85 patients with HNSCC.

Variable	Category	Patients n (%)
Age	Median (Range; IQR)	60.0 (30.0-85.4; 14.0)
	< 60	42 (49.4)
	≥ 60	43 (50.6)
Gender	Male	70 (82.4)
	Female	15 (17.6)
Tumor Site	Oral cavity	20 (23.5)
	Oropharynx	22 (25.9)
	Larynx	22 (25.9)
	Hypopharynx	21 (24.7)
p16	Negative	77 (90.6)
	Positive	8 (9.4)
T stage	T1	8 (9.4)
	T2	31 (36.5)
	T3	21 (24.7)
	T4	25 (29.4)
N stage	N0	19 (22.4)
	N1	4 (4.7)
	N2	62 (72.9)
	N3	0 (0)
Grading	1	3 (3.5)
	2	36 (42.4)
	3	46 (54.1)
R stage	0	43 (50.6)
	1	32 (37.6)
	x	10 (11.8)
UICC stage	I	3 (3.5)
	II	3 (3.5)
	III	4 (4.7)
	IVa	75 (88.2)
	IVb	0 (0)
Surgery	Yes	82 (96.5)
	No	3 (3.5)
Adjuvant treatment	None	16 (18.8)
	RTx	37 (43.5)
	CRTx	32 (37.6)
	CTx	0 (0)

HNSCC, Head and Neck Squamous Cell Carcinoma; IQR, interquartile range; T stage, size of tumors staged 1-4; N stage, presence of lymph node metastases, staged 0-3; R stage, indicates rest of tumor in resected tumor margins, 0 – free of tumor, 1 – tumor *in situ*, x – cannot be clarified pathologically; UICC, Union for International Cancer Control; RTx, Radiation only; RCTx, Radiochemotherapy; CTx, Chemotherapy only.

### 2.2 Multicolor immunofluorescence staining

**Figure 1:**
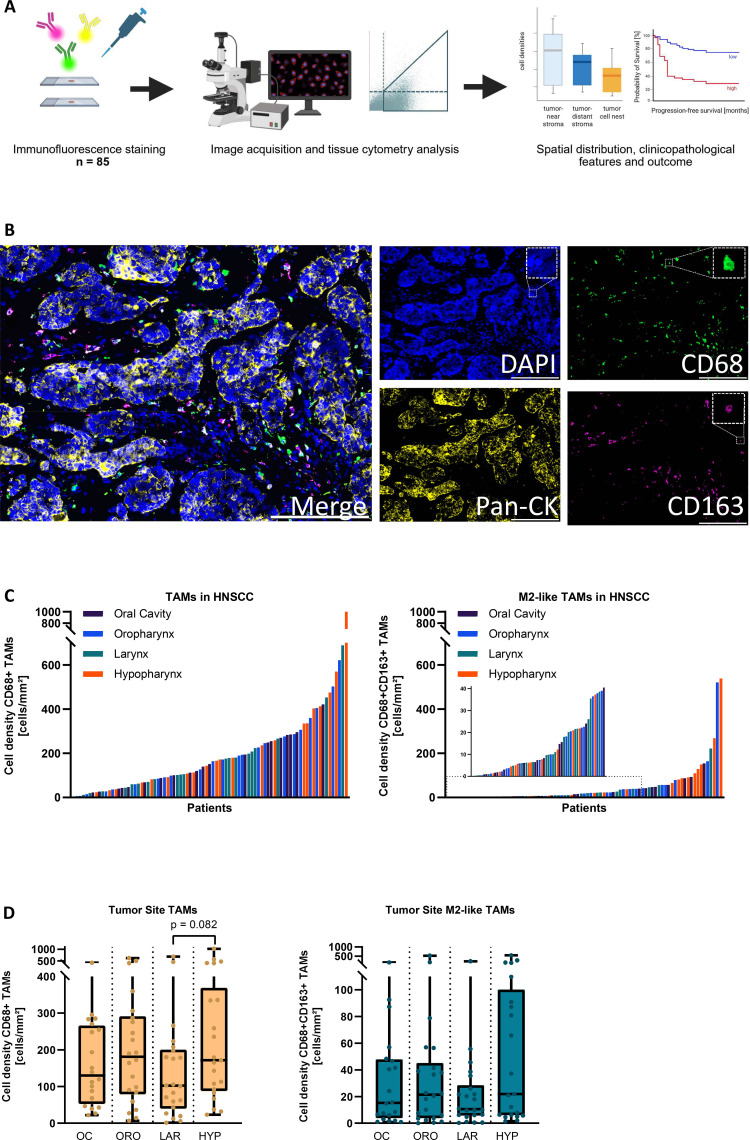
Detection and quantification of TAMs and M2-like TAMs in head an neck squamous cell carcinoma (HNSCC). **(A)** Study workflow: multicolor immunofluorescence staining followed by high-resolution automated multiple image alignments of whole-tissue sections, semiautomated detection and quantification at single-cell level, and uni- and multivariate survival analysis. **(B)** Representative multicolor fluorescent images of tumor-associated macrophages (TAMs, CD68+) and M2-like TAMs (CD68+CD163+) staining in HNSCC. Nuclei stained with DAPI in blue. pan-cytokeratin (Pan-CK) determined epithelial tumor cell nests in yellow. TAMs and M2-like TAMs stained with CD68 in green and CD163 in purple. scale bar: 200 µm. **(C)** Waterfall plot showing ranked cell densities of TAMs (CD68^+^) and M2-like TAMs (CD68^+^CD163^+^) across the total cohort of 85 HNSCC cases, annotated by tumor site. This representation highlights the broad inter-patient variability, distribution range, and overlap of TAM infiltration across different tumor sites. **(D)** TAM (CD68^+^) and M2-like TAM (CD68^+^CD163^+^) densities in the total cohort, stratified by tumor site and presented as boxplots to facilitate group-wise comparison. OC, Oral Cavity carcinoma; ORO, Oropharyngeal carcinoma; LAR, Laryngeal carcinoma; HYP, Hypopharyngeal carcinoma.

Multicolor immunofluorescence staining was performed on acetone-fixed cryosections (5-7 μm) of whole-tissue slices of HNSCC specimens as reported previously (**Figure 1A**) (36). To quantify TAM and M2-like TAM subpopulations, a combination of primary antibodies specific for CD68 (mouse anti-human, AF488-coupled, Santa Cruz #sc-20060 AF488) and CD163 (rabbit anti-human, CD163, abcam #ab182422) were applied. CD68 is a pan-macrophage marker and is highly expressed in the monocyte lineage (20). To identify TAMs with a pro-tumoral M2-like phenotype, we co-stained for the scavenger receptor CD163, which is predominantly expressed in the monocyte lineage, and a well-accepted marker for M2-like polarized TAMs (21, 37). Further, an antibody specific for pan-cytokeratin (mouse anti-human, eFluor570-coupled, ThermoFisher #41-9003-82) was used to identify epithelial tumor cell nests within HNSCC tissues. Antibody diluent (Dako) was used to dilute primary antibodies. For CD163 staining, primary antibody was coupled to secondary goat anti-rabbit AlexaFluor647 antibody, which was diluted with DPBS-containing DAPI (1:1,000 dilution; ThermoFisher Scientific) to stain nuclei. Primary and secondary antibodies were incubated for 1h at room temperature, followed by three washing steps with DPBS-Tween (0.05%). Human tonsil tissue and isotype-matched antibodies served as positive and negative controls, respectively.

### 2.3 Tissue cytometry-based image analysis

For tissue cytometry-based image analysis, high-resolution automated multiple image alignments of whole-tissue sections were acquired using a 20× objective on an Olympus IX51 microscope equipped with a XM10 Camera (Olympus). For image acquisition, the Olympus CellSens Dimension Software (version 1.9) was used. Subsequently, semiautomated detection and quantification at a single-cell level was conducted using acquired microscopic images by help of the StrataQuest Software (version 5.0.1, TissueGnostics GmbH). A hierarchical gating strategy was applied to conduct automatic nuclei-based detection and context-based quantification of TAM and M2-like TAM infiltration as well as cytokeratin-positive tumor cells by immunofluorescence markers. Regions of interest were manually defined depending on histology and quality of the section to exclude adjacent normal non-tumor tissue or necrotic areas. Regions of interest were drawn in the slide overview using software-based mark-up tools. Quantification was solely performed in areas with high tumor cell content (≥ 60%) and, if present, necrotic areas were generously excluded. Automatically detected cells were visualized in scattergrams and gated according to defined gating schemes for the expression of nucleic and cell surface markers. Cutoff between positive- and negative-gated cells was validated by backward gating. To enable robust and reliable cell quantification, strict parameters by means of nuclear size, staining intensity, and background threshold were defined. Cell nuclei were detected based on DAPI staining and used as origin to generate a growing mask over the cytoplasm to the cell membrane. Based on this mask, TAMs were analyzed regarding cell surface expression of CD68. M2-like TAMs were defined by additional cell surface expression of CD163. Tumoral and stromal compartments were distinguished by cell surface expression of cytokeratin and analyzed as reported earlier (36). To assess regional variability in stromal HNSCC tissue specimens, we analyzed different stromal areas: (1) stromal compartments within 100 μm distance from tumor cell nests in the following named as “tumor-near stroma”, and (2) stromal compartments with > 100 μm distance from tumor cell nests in the following named as “tumor-distant stroma”. Tissue areas analyzed and cell densities are summarized in **Supplementary Table 2**.

### 2.4 Immunohistochemical staining

Immunohistochemical staining of HNSCC tissue specimens was conducted to determine p16-status. P16-status was used as surrogate parameter for an infection with HPV. The primary p16-specific antibody (mouse, 1:50, 550834, BD Pharmingen) and an isotype control (human, 1:160, ab91353, Abcam) were diluted with Antibody Diluent (Dako) and incubated at 37 C for 45 min. Vectastain Elite ABC Kit (HRP) (mouse, Vector Laboratories) detected primary antibody binding and the enzymatic staining reaction was stopped after 30 min. Hematoxylin was used to counterstain nuclei. An HPV+ HNSCC tissue with known p16-positive staining served as positive control.

### 2.5 Statistical analysis and visualization

Data visualization and univariate analysis were conducted using GraphPad Prism (Version 10.4.0). Multivariate analysis was performed using the software R (Version 4.4.1). Data are presented in boxplots, showing the median, interquartile range, and whiskers from minimum to maximum with all data points plotted. To allow differentiation between low and high ages or densities, the median value was used for each of these variables to indicate low or high levels. For paired analyses, the Wilcoxon matched-pairs signed rank test was conducted. For unpaired testing, the Mann-Whitney U test was applied. Progression-free survival (PFS) was defined as interval from time of surgery until tumor progression. Overall survival (OS) was defined as interval from time of surgery until patient death. Kaplan-Meier curves were applied to present univariate survival data with p-values calculated by the log rank test. Univariate analyses were performed for all clinically relevant variables. Variables showing a statistical association in univariate analysis (p < 0.1) were subsequently included in the multivariate analysis using a Cox proportional hazards model. This stepwise approach was applied to reduce the risk of overfitting, particularly given the limited cohort size. To enhance statistical power and precise data reporting, also continuous variables have been reported in the multivariate analysis using a Cox proportional hazard model. P-values < 0.05 were considered significant: * p < 0.05; ** p < 0.01; *** p < 0.001. For better transparency and understanding of the data, we also reported on non-significant p-values.

## 3 Results

### 3.1 Highly variable TAM and M2-like TAM infiltration at distinct HNSCC tumor sites

To study TAM and M2-like TAM densities and their spatial distribution in HNSCC, we performed multicolor immunofluorescence stainings in a clinically well-annotated treatment-naïve cohort of n = 85 HNSCC patients (**Table 1**). Median age of patients was 60 years at time of diagnosis with a female-to-male ratio of 1.0 to 4.7. The study cohort primarily comprised advanced HNSCC tumors of UICC-stage IVa (n = 75; 88.2%), including all four T-stages and N-stages ranging from N0 to N2. Postoperative adjuvant treatment was performed on 77.6% of patients in the study cohort, with 43.5% receiving radiation alone and 34.1% receiving chemoradiotherapy. Of the HNSCC patients included in the study, 90.6% (n = 77) were p16-negative and 9.4% were p16-positive (n = 8). Six of eight p16-positve HNSCC specimens were located in the oropharynx, and two in the oral cavity. Different HNSCC sites (oral cavity, oropharynx, larynx, hypopharynx) were almost equally distributed in the study cohort (**Table 1**). M2-like TAMs were identified using a combination of CD68, as general TAM marker, and the scavenger receptor CD163 as a marker for the pro-tumoral and immunosuppressive M2-like TAM phenotype (16, 17, 20), while cytokeratin staining was used to label tumor cells (**Figure 1B**). Our analysis revealed a large heterogeneity for TAM- and M2-like TAM densities at all tumor sites of our study cohort ranging from 1.5 to 1,018.0 cells/mm² for TAMs and from 0.0 to 538.4 cells/mm² for M2-like TAMs (**Figure 1C**). Highest median numbers of TAMs and M2-like TAMs were observed in oropharynx (TAMs: median 181.1 cells/mm²; range: 7.0-620.9 cells/mm²; M2-like TAMs: 21.4 cells/mm², range: 0.0-521.1 cells/mm²) and hypopharynx carcinoma (TAMs: median: 171.5 cells/mm², range: 23.4-1,018 cells/mm²; M2-like TAMs: median: 21.9 cells/mm²; range: 1.5-538.4 cells/mm²; **Figure 1D**). However, highest variability and highest overall numbers of M2-like TAMs were observed in a small subgroup of hypopharynx carcinomas (**Figures 1C, D**).

When looking at the impact of TAMs and M2-like TAMs on survival of the whole study cohort, high TAM densities did not have a significant effect on PFS and OS (**Supplementary Figure 1A**), whereas high numbers of infiltrating M2-like TAMs significantly decreased PFS (p < 0.05), but not OS (**Supplementary Figure 1B**).

In summary, we observed a large heterogeneity in the frequencies of TAMs and M2-like TAMs in HNSCC. Especially, higher numbers of pro-tumoral M2-like TAMs were associated with an inferior PFS and were more often found in a subgroup of hypopharyngeal carcinoma.

### 3.2 P16-negative HNSCCs show higher TAM and M2-like TAM densities in all tumor areas analyzed

**Figure 2:**
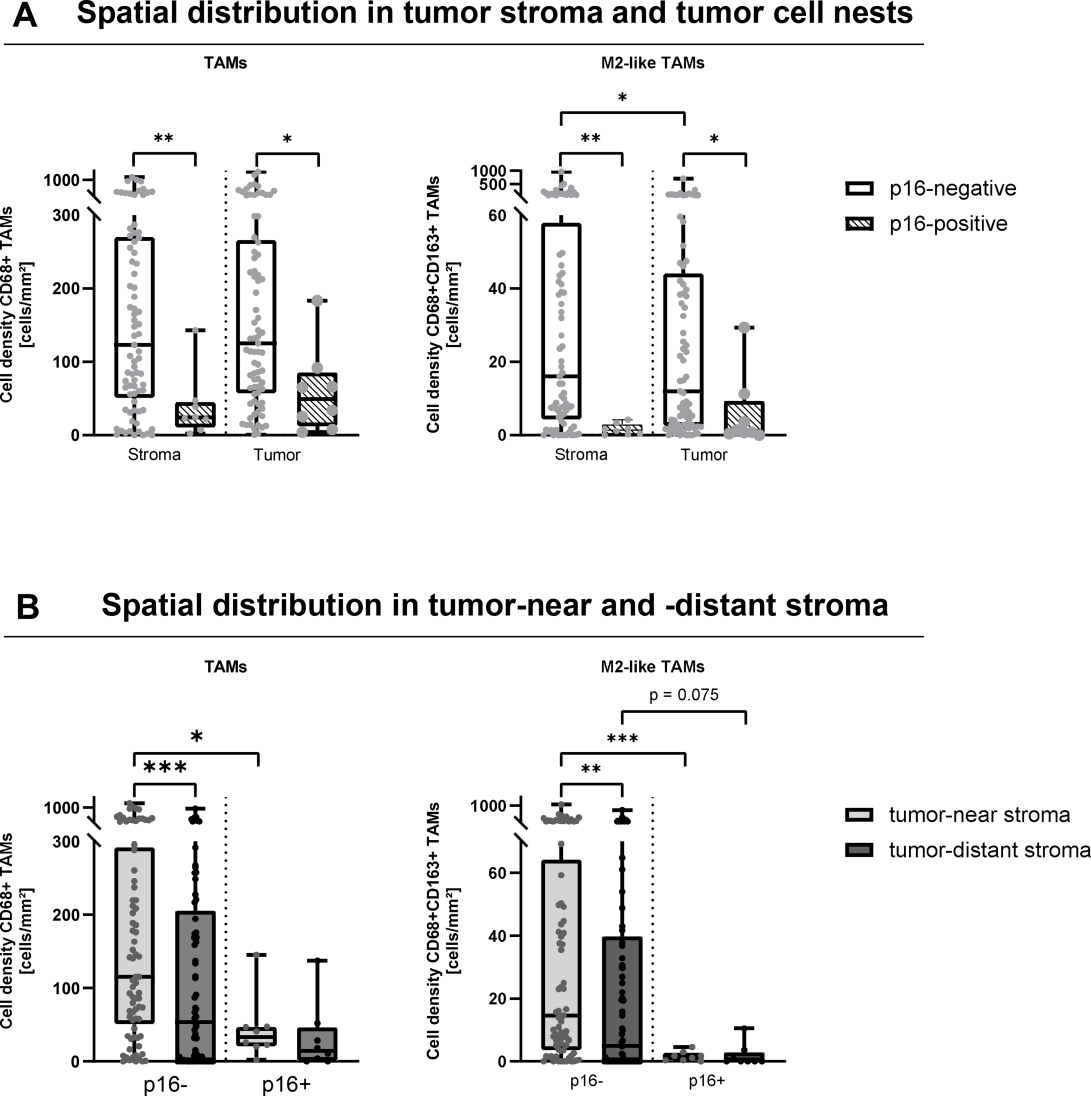
P16-status and spatial distribution of TAMs and M2-like TAMs. **(A)** CD68+ and CD68+CD163+ (M2-like) tumor-associated macrophage (TAM) infiltration into tumor stroma and tumor cell nests of p16-negative (white boxes, n = 77) and p16-positive (shaded boxes, n = 8) head and neck squamous cell carcinomas (HNSCC). **(B)** CD68+ and CD68+CD163+ (M2-like) TAM infiltration into tumor-near stroma (bright grey) and tumor-distant stroma (dark grey) of p16-negative (n = 77) and p16-positive (n = 8) HNSCCs. *p < 0.05; **p < 0.01; ***p < 0.001.

To learn more about the spatial distribution of TAMs, we next analyzed their frequencies in the tumor stroma and tumor cell nests including the distance to tumor islands. Furthermore, we included the p16-status, because it has emerged as an important prognostic confounder in HNSCCs (38). In line with this assumption, univariate survival analysis revealed an almost significant inferior PFS in p16-negative specimen as compared to p16-positive tumors (23.2. months vs. 123.2 months, p = 0.066), but no influence on OS in this study cohort (**Supplementary Figure 2**). Further, our immune cell infiltration analysis showed substantial differences in the spatial distribution of TAMs and M2-like TAMs between p16-negative (n = 77) and p16-positive (n = 8) HNSCCs. In p16-negative tumors, median TAM and M2-like TAM infiltration was significantly higher in the tumor stroma (TAMs: 123.1 vs. 23.9 cells/mm²; M2-like TAMs: 16.1 vs. 1.3 cells/mm²) and tumor cell nests (TAMs: 125.2 vs. 49.5 cells/mm²; M2-like TAMs: 12.0 vs. 1.1 cells/mm²) compared to p16-positive tumors (**Figure 2A**). Moreover, in p16-negative tumors, M2-like TAMs were significantly enriched in the tumor stroma as compared to tumor cell nests. Interestingly, in p16-negative tumors highest TAM and M2-like TAM frequencies were found in the tumor-near stroma, whereas in p16-positive tumors low TAM and M2-like TAM densities did not further decline in the tumor-distant stroma (**Figure 2B**).

Taken together, our analysis revealed that the p16-status affects the density and spatial distribution of TAMs and M2-like TAMs, with significantly higher densities observed in p16-negative specimens. Furthermore, the stroma of p16-positive HNSCC was particularly characterized by a reduced number of M2-like TAMs.

### 3.3 Stromal and tumoral M2-like TAM infiltration differs between HNSCC tumor sites, age and gender

Based on the observation that the small subset of p16-positive tumors showed an up to 5-fold lower TAM and 12-fold lower M2-like TAM infiltration and were associated with better PFS (**Figure 2**, **Supplementary Figure 2**), these cases were excluded from our subsequent analysis.

To further elucidate the spatial distribution in p16- HNSCC and their dependence on clinicopathological parameters, we investigated tumor stroma and tumor cell nests separately in our tumor specimens, using cytokeratin to demarcate tumor islands (20, 21, 35, 39). Likewise, we performed a more in-depth spatial analysis of the tumor stroma by distinguishing between immune cell densities in tumor-near and tumor-distant stroma. **Supplementary Table 2** summarizes the analyzed tissue areas and the median immune cell densities.

**Figure 3:**
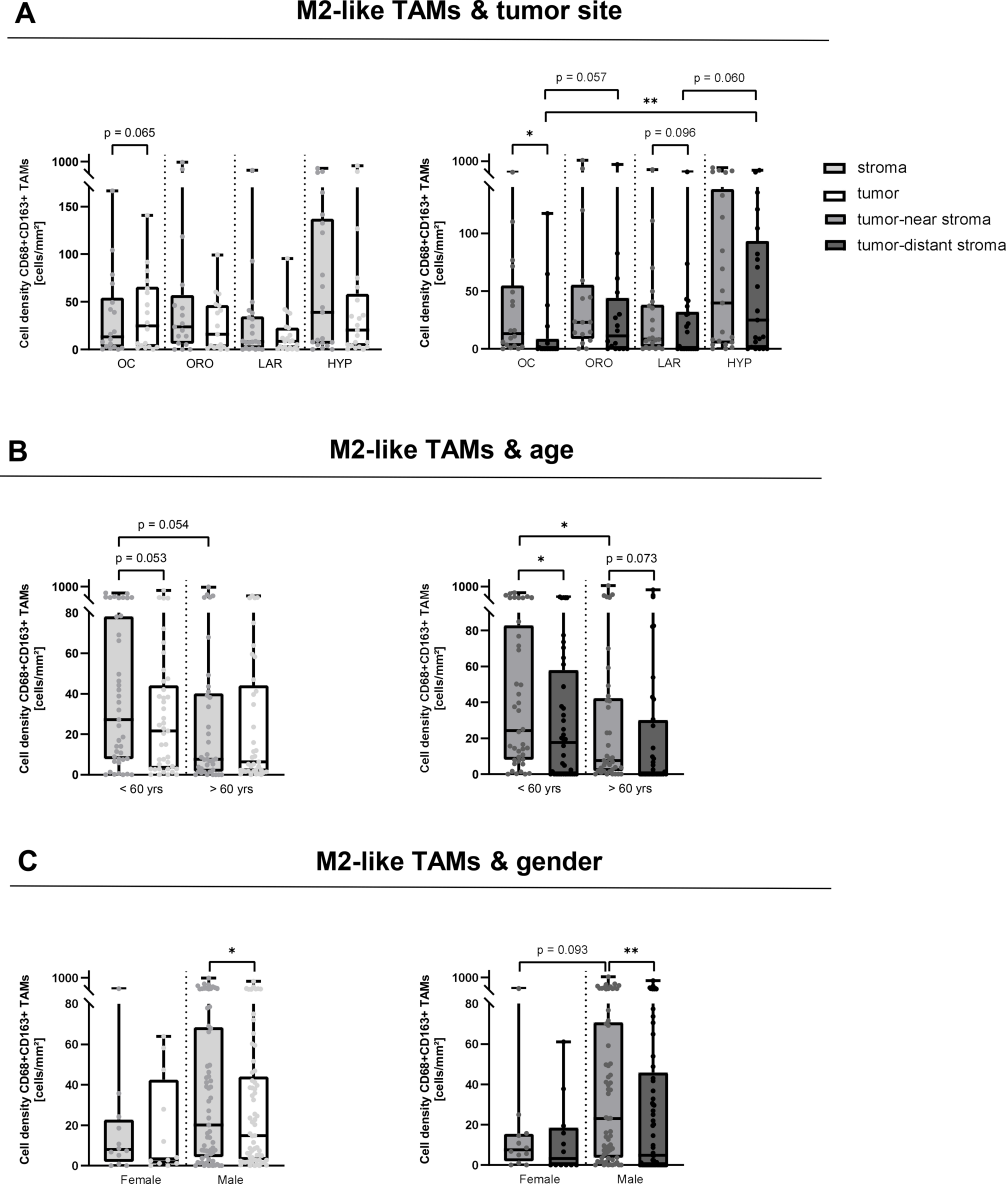
Spatial distribution of M2-like TAMs related to clinicopathological features. Infiltration of CD68+CD163+ tumor-associated macrophages (M2-like TAMs) into tumor stroma (bright grey) and tumor cell nests (white) (left panel) and into tumor-near (middle grey) and tumor-distant stroma (dark grey) (right panel) related to tumor site **(A)**, age **(B)**, and gender **(C)** in p16-negative head and neck squamous cell carcinomas (n = 77). *p < 0.05; **p < 0.01. OC, Oral Cavity carcinoma; ORO, Oropharyngeal carcinoma; LAR, Laryngeal carcinoma; HYP, Hypopharyngeal carcinoma.

First, we examined immune cell densities at different tumor sites and found no significant differences in TAM (**Supplementary Figure 3A**) and M2-like TAM (**Figure 3A**) densities between the localizations and their spatial distribution in tumor stroma and tumor cell nests. However, hypopharyngeal carcinomas tended to have 1.6 to 4.7-fold higher median stromal M2-like TAM densities than the other tumor sites (oral cavity: 13.4; oropharynx carcinoma: 23.6; larynx carcinoma: 8.2; hypopharynx carcinoma 38.9 cells/mm²). Moreover, while in the hypopharynx, oropharynx and larynx both immune cell densities were lower in tumor cell nests, in the oral cavity the opposite phenomenon was observed for M2-like TAMs and TAMs almost reaching a level of significance for M2-like TAMs (p = 0.065; **Figure 3A**, **Supplementary Figure 3A**). Noteworthy, laryngeal carcinomas had the lowest stromal and intratumoral infiltrations of M2-like TAMs of all tumor sites (tumor stroma: 8.2 cells/mm², tumor cell nest: 8.6 cells/mm²). This is in line with the observation that, in the whole tumor tissues, laryngeal carcinoma also presented with the lowest densities of TAMs and M2-like TAMs compared to the other tumor sites (**Figure 1D**).

When looking at the distance of stromal TAMs and M2-like TAMs to tumor cell nests, we observed a decrease in both immune cell densities in the tumor-distant stroma of all tumor localizations. This was significant for TAMs in all tumor sites, but less pronounced for M2-like TAMs and, except for the oral cavity, did not reach a level of significance for M2-like TAMs (**Figure 3A**, **Supplementary Figure 3A**). Nevertheless, highest infiltration of both immune cell types in the tumor-distant stroma was observed for the hypopharynx.

When we examined the age-related spatial immune cell infiltration, we observed a higher prevalence of TAMs and M2-like TAMs in younger patients (median split, 60 years) and in all tumor compartments analyzed (tumor cell nests, entire stroma, tumor-near and tumor-distant stroma, **Figure 3B**, **Supplementary Figure 3B**). This particularly applied to TAM and M2-like TAM densities in the tumor-near stroma as compared to the tumor-distant stroma.

Further, gender-associated aspects were analyzed. Here, M2-like TAM densities were overall higher in HNSCC tissues of male patients with the highest density in the tumor-near stroma and a significant decrease in the tumor-distant stroma (**Figure 3C**) (tumor stroma: 20.2 vs. 8.0 cells/mm², p = 0.128; tumor cell nests: 14.9 vs. 3.4 cells/mm², p = 0.169). As for gender-related TAM densities, differences were minor, independent of the tumor compartment analyzed. Only in the tumor-distant stroma of male patients we observed a significant decrease in TAM densities (**Supplementary Figure 3C**).

Altogether, hypopharyngeal carcinomas showed the highest densities of TAMs and M2-like TAMs in all tumor areas analyzed, whereas tumors of the oral cavity presented with highest infiltration of tumor cell nests even exceeding that of the adjacent tumor stroma in contrast to the other tumor sites. Moreover, especially younger and male patients exhibited greater M2-like TAM infiltration in the tumor-near stroma than older and female patients, respectively.

### 3.4 Tumor-near stroma of nodal positive HNSCCs shows highest M2-TAM densities

Further, we investigated the influence of tumor stage, T-stage and nodal status on TAM and M2-like TAM densities to elucidate their role in advanced tumors.

When looking at the infiltration of TAMs into the whole tissue as compared to the specific tumor and stroma infiltration, there were no differences in TAM densities related to tumor stage and T-stage (**Supplementary Figures 4A, B**). However, TAM infiltration significantly decreased in the tumor-distant stroma of higher stages and all T-stages (**Supplementary Figures 4A, B**).

Interestingly, nodal-positive HNSCCs had a higher density of TAMs, nearly reaching statistical significance when analyzing the whole tissues (p = 0.058, **Supplementary Figure 4C**). In addition, nodal-positive HNSCCs presented with a significantly increased infiltration of the stroma that was highest in the tumor-near stroma (**Supplementary Figure 4C**).

**Figure 4:**
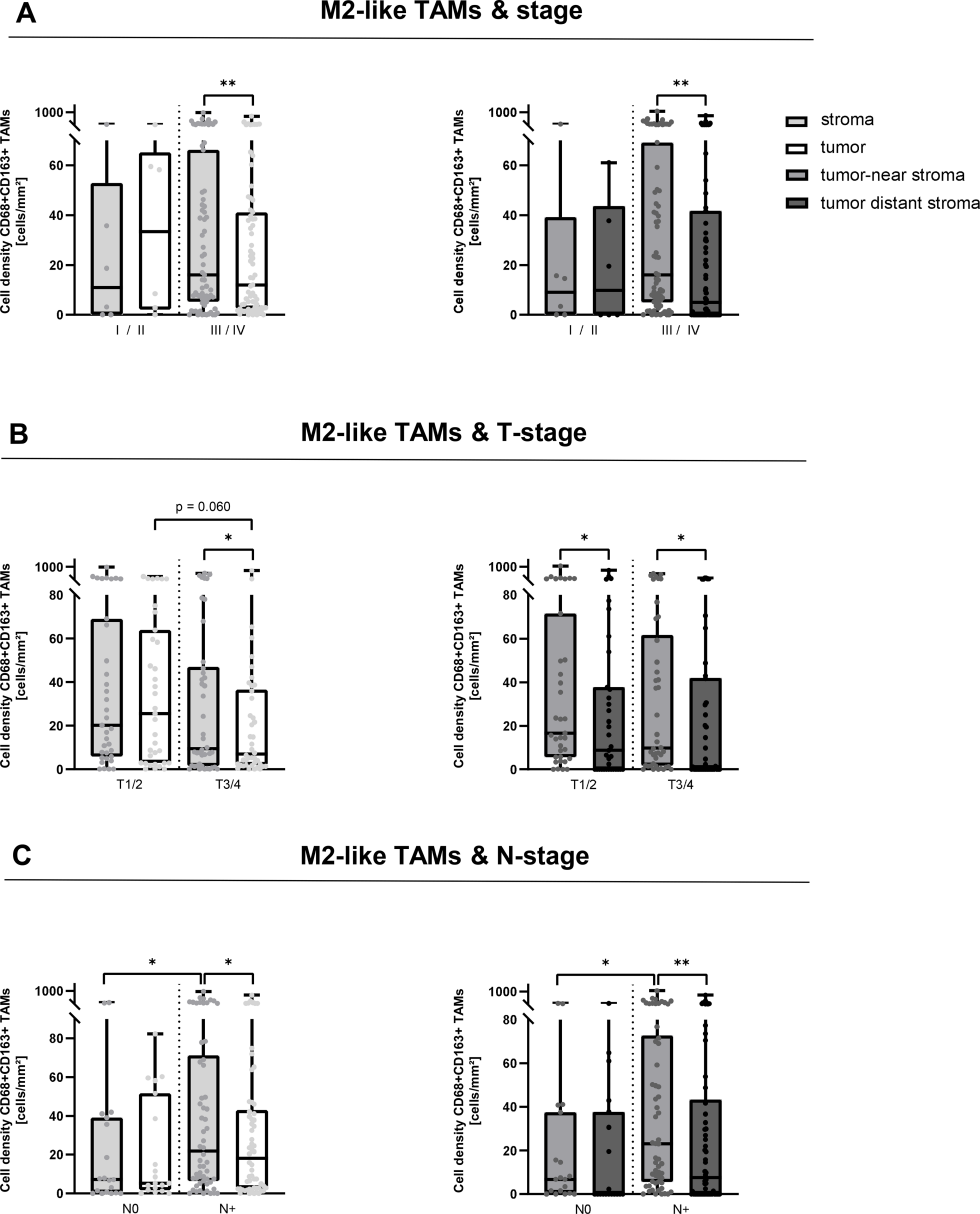
Spatial distribution of M2-like TAMs related to tumor stages. Infiltration of CD68+CD163+ tumor-associated macrophages (M2-like TAMs) into tumor stroma (bright grey) and tumor cell nests (white) (left panel) and into tumor-near stroma (middle grey) and tumor-distant stroma (dark grey) (right panel) related to stage **(A)**, T-stage **(B)**, and N-stage **(C)** in p16-negative head and neck squamous cell carcinomas (n = 77).*p < 0.05; **p < 0.01.

When looking at M2-like TAM densities in the whole tissues, their numbers were slightly decreased in higher-staged tumors, significantly decreased in tumors categorized as T3/4 compared to tumors categorized as T1/2 and slightly higher in nodal-positive-HNSCCs (**Supplementary Figures 5A–C**). When analyzing the spatial distribution of different tumor areas depending on stage, T-stage and nodal status, we observed significantly more M2-like TAMs in the stroma of higher-staged tumors as compared to their tumor cell nests, with the highest infiltration occurring in the tumor-near stroma (**Figure 4A**). Interestingly, tumors categorized as T3/4 seemed to have a lower M2-like TAM infiltration in the tumor stroma and even more in tumor cell nests, whereas their number further decreased in the tumor-distant stroma independent of the T-stage (**Figure 4B**). Noteworthy, the opposite was seen for nodal-positive tumors, where M2-like densities were significantly higher in the tumor stroma of nodal-positive HNSCC as compared to corresponding tumor cell nests (**Figure 4C**). Moreover, the median stromal density of M2-like TAMs in nodal-positive HNSCCs was significantly higher than in those with N0 (21.9 vs. 7.1 cells/mm², p < 0.05) (**Figure 4C**). Further, M2-like TAM densities were lower in the tumor-distant stroma compared to tumor-near stroma. As seen before for TAM densities (**Supplementary Figures 4A, C**), we observed the highest densities of M2-like TAMs in the tumor-near stroma of metastasized high-staged HNSCCs (**Figures 4A, C**).

Taken together, advanced tumors and especially nodal-positive HNSCC displayed a significantly higher density of M2-like TAMs in the tumor-near stroma as compared to other tumor compartments and nodal-negative HNSCC specimen.

### 3.5 M2-like TAM densities have a higher impact on survival than tumor site and p-16 status

Finally, we performed univariate and multivariate survival analyses to assess the impact of TAM and M2-like TAM infiltration on patient outcomes.

**Figure 5:**
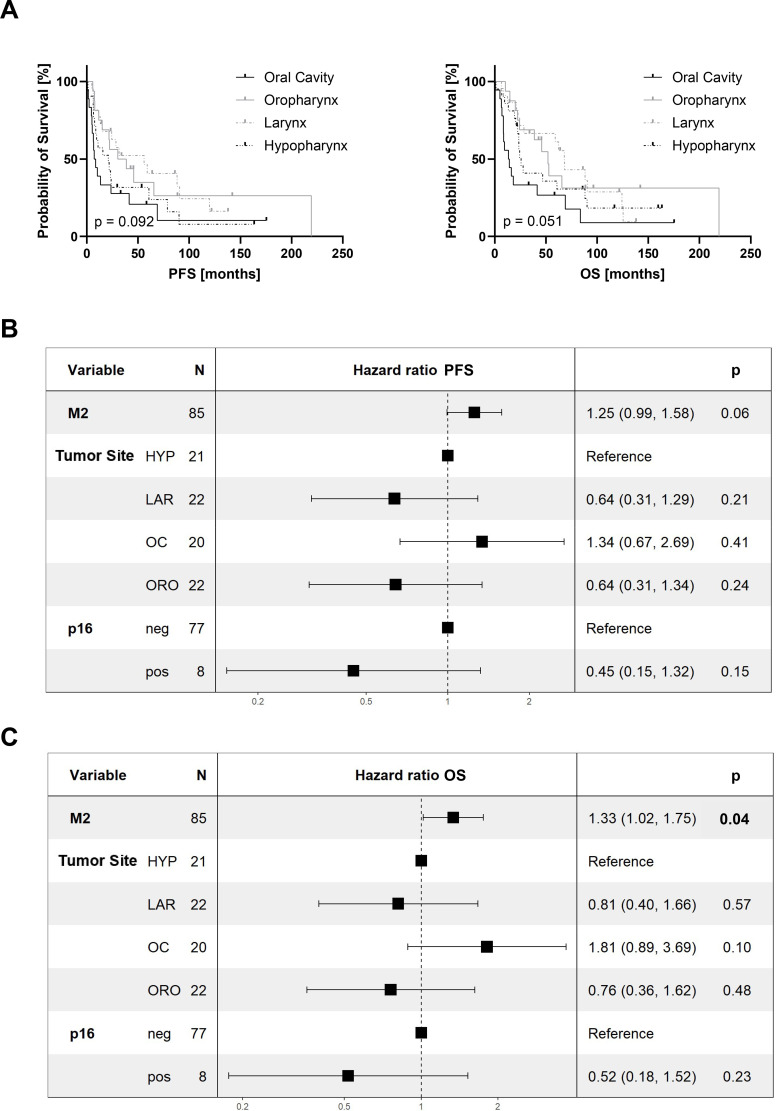
Univariate and multivariate survival analysis. **(A)** Progression-free (PFS) and overall survival (OS) depending on tumor site in p16-negative head and neck squamous cell carcinomas (HNSCC, n = 77). Multivariate analyses of **(B)** progression-free survival (PFS) and **(C)** overall survival (OS) including 85 HNSCCs. p: p-value; n: Absolute number of included HNSCCs; M2: CD68+ CD163+ Tumor-associated macrophages; HYP, Hypopharyngeal carcinoma; LAR, Laryngeal carcinoma; OC, Oral Cavity carcinoma; ORO, Oropharyngeal carcinoma; neg: p16-negative; pos: p16-positive. Bold: significant p-value < 0.05.

Whereas there were no significant differences in the univariate survival analysis for PFS (**Supplementary Figure 6**) and OS (**Supplementary Figure 7**) for several clinicopathological features including patient age, gender, tumor stage, T-stage, N-stage, and adjuvant treatment for p16-negative specimens (n = 77), tumor site showed a tendency to influence PFS (p = 0.092) and OS (p = 0.051) with the best outcome for larynx carcinoma and the most inferior outcome for patients suffering from p16-negative tumors of the oral cavity (**Figure 5A**) (36). Therefore, we excluded patient age, gender, tumor stages, and adjuvant therapies from the multivariate analysis and included only tumor sites, p16-status, as well as TAM and M2-like TAM densities in our model. Whereas tumor site and p16-status did not impact PFS, total M2-like TAM densities presented with a Hazard Ratio of 1.25 (95%-Confidence interval (CI): 0.99-1.58, p = 0.06) and thus, showing a trend to negatively influence PFS (**Figure 5B**). Moreover, in the OS analysis total M2-like TAM densities had a significant Hazard Ratio of 1.33 (95%-CI: 1.02-1.75; p = 0.04), negatively affecting patient outcome, which was not observed for the tumor site and p16-status (**Figure 5C**). However, total TAM densities presented no significant survival associations (**Supplementary Figures 8A, B**).

In summary, while most clinicopathological features had no impact on patient survival, high M2-like TAM densities turned out to be an independent prognostic marker for poor OS and thus had a higher influence on survival than known prognostic confounders such as tumor site or p16-status in our multivariate model.

## 4 Discussion

TAMs have been shown to be the most frequent population of immune cells in the TME of many solid cancers, where especially M2-like activated TAMs are known to support tumor growth and disease progression (18, 20, 21, 23, 34). To gain a deeper insight into the role of TAMs and their subpopulations in HNSCC, we quantified TAMs and M2-like TAMs in a large cohort of treatment-naïve HNSCC tumors. This revealed an infiltration gradient that culminated in the highest density of M2-like TAMs in the tumor-near stroma of advanced nodal-positive p16-negative HNSCC. Furthermore, we observed a higher prevalence in younger and male patients and in a subgroup of patients suffering from hypopharyngeal carcinomas. Moreover, high levels of M2-like TAMs were associated with inferior OS in these patients, independent of other known prognostic confounders. This even exceeded the impact of the p16-status and the tumor site.

As a major strength, the current study provides a comprehensive overview on the spatial distribution of macrophages in the tumor stroma and tumor cell nests using a workflow allowing for the objective single-cell based quantification of large tissue sections. Moreover, it adds important knowledge as it investigated not only the role of TAMs in HNSCC but also of pro-tumoral M2-like TAMs, included considerable case numbers of all major tumor sites, and considered their role in p16-positive and -negative HNSCC in a well-annotated study cohort of 85 patients. This is in contrast to former studies, where a less detailed quantification was performed mostly on a limited number of small microscopical section and by the help of a less resolved semi-quantitative score-based assessment revealing partially controversial results (24, 30, 35, 39). With the technological advances of the present study, we were able to dissolve some of these inconsistencies by a more precise and detailed quantification workflow and by analyzing a substantial number of cases from all major tumor sites.

As one of our main observations we found significantly higher stromal densities of M2-like TAMs in advanced tumors (UICC-stage III/IV; T3/4; N+) as compared to adjacent tumor-cell nests, particularly in the tumor-near stroma of nodal-positive as compared to nodal-negative HNSCCs. This strongly suggests that abundant M2-like TAMs, particularly in the tumor stroma, might indicate more advanced HNSCCs.

Another important contribution to the existing knowledge consists of the detailed examination of the tumor stroma by distinguishing between tumor-near and -distant stromal compartments. Here, our study showed that the density of M2-like TAMs already decreases significantly at a distance of only 100 µm next to tumor islands as compared to the tumor-near stroma. Although Balermpas et al. investigated M2-like TAM densities in the tumor, in the tumor stroma, and in the tumor periphery (24), and Ou et al. analyzed the intraepithelial and stroma macrophage densities (35), none of these studies described a similar density gradient of M2-like TAMs from tumor cell nests to tumor-near and tumor-distant stroma as their analysis was based on a semi-quantitative workflow.

As a clinically and translationally relevant impact of our study, we not only confirmed the assumed prognostic value of high densities of M2-like TAMs for PFS in a univariate survival analysis (24, 35), but we also showed in a multivariate analysis that high M2-like TAM densities represent an independent prognostic marker for inferior overall survival in HNSCC patients. This was also suggested by others, although they analyzed a less balanced cohort regarding tumor sites (24, 35). Accordingly, the identification of M2-like TAMs as a prime target in HNSCC could be of particular importance to improve the therapy of clinically aggressive HNSCC subtypes by addressing M2-like macrophages as an alternative treatment option (20, 21, 27, 40). For instance, there is increasing evidence that eradicating M2-like TAMs by targeting the colony-stimulating factor 1-/-receptor or even promoting a phenotypic switch from pro-tumoral M2-like TAMs to anti-tumoral M1-like TAMs could result in a more immune-active TME (21, 23, 40).

HPV-driven HNSCC seem to be immunologically more active due to the expression of viral neoantigens and higher levels of cytotoxic T-cells and T helper cells (14, 15, 30, 35, 36). However, several studies investigating the presence of TAMs and pro-tumoral M2-like TAMs in HPV-positive and -negative HNSCC came to contradictory results, describing either an unexpected higher TAM infiltration in HPV-positive HNSCC (39) or a higher infiltration of M2-like TAMs in HPV-negative tumors (35). In our study, the thorough quantification of TAMs and M2-like TAMs in whole tissue sections gave strong support for the latter observation and revealed a lower prevalence of TAMs and even more of M2-like TAMs in HPV-positive HNSCCs. Furthermore, despite lower TAM and M2-like TAM levels in p16-positive tumors, in good agreement with others, both cell types showed a higher presence in tumor cell nests as compared to the stroma (30, 35).

Nevertheless, our study has some limitations, including the relatively low number of p16-positive HNSCC cases compared to HPV-negative tumors. This imbalance, which is commonly reported in similar studies, may limit the statistical power of analyses performed on the complete cohort (23, 35). Furthermore, in the current study, the total cohort size consisted of 85 patients, which should be further increased in future studies to substantiate our associations with clinical parameters and tumor localization-dependent findings. Nevertheless, different tumor sites were equally represented in our study cohort, which represents an advance as compared to previous studies focusing either solely on HNSCC of the oral cavity (16, 21, 32, 34) or on different tumor sites, but with an uneven distribution of distinct tumor sites within their cohort (39). Further, we acknowledge that TAMs comprise a highly heterogeneous population, extending beyond the simplified M2-like classification. While our study was designed to investigate immunosuppressive TAMs due to their well-established role in tumor progression and immune evasion (18, 26, 41), this approach does not capture the full spectrum of TAM diversity. Addressing TAM heterogeneity in HNSCC represents an important objective for future studies.

In conclusion, this study expands our current understanding of the role of TAMs and their subpopulations in HNSCC. Especially pro-tumoral M2-like TAMs seem to play a major role, as they were not only highly enriched in the tumor-near stroma of HNSCC, particularly in hypopharynx and nodal-positive tumors, but higher densities of M2-like TAMs were also associated with inferior overall survival in our study cohort, independent of other prognostic confounders, such as p16 status and tumor site. Notably, p16-positive HNSCCs displayed comparably low densities of M2-like TAMs, which may contribute to the more favorable prognosis of patients typically observed in HPV-driven carcinomas.

## Data Availability

The original contributions presented in the study are included in the article/**Supplementary Material**. Further inquiries can be directed to the corresponding author.

## Supplementary material

Supplementary Table 1 Patient characteristics of 77 patients with p16-negative HNSCC.

Supplementary Table 2 Tissue area analyzed and densities of TAMs and M2-like TAMs.

Supplementary Figure 1 Survival analysis in the total study cohort in relation to TAM- and M2-like TAM infiltrations.

Supplementary Figure 2 Univariate survival analysis and p16-status.

Supplementary Figure 3 Spatial distribution of TAMs related to clinicopathological features.

Supplementary Figure 4 Spatial distribution of TAMs related to clinicopathological features.

Supplementary Figure 5 CD68+CD163+ M2-like TAM infiltration of whole tumor tissue in p16-negative head and neck squamous cell carinomas (n = 77) in relation to stage **(A)**, T-stage **(B)**, and N-stage **(C)**.

Supplementary Figure 6 Progression-free survival (PFS) analysis of patients with p-16 negative head and neck squamous cell carcinomas (HNSCCs) related to clinicopathological features.

Supplementary Figure 7 Overall survival (OS) analysis of patients with p-16 negative head and neck squamous cell carcinomas (HNSCCs) related to clinicopathological features.

Supplementary Figure 8 Multivariate analysis of total TAM densities including the covariates tumor site, and p16-status.
