# Supplementary material for: Spatial distribution and prognostic value of tumor-associated macrophages in head and neck squamous cell carcinomas
Source: Front Oncol. 2026 May 8;16:1806162. doi: 10.3389/fonc.2026.1806162 (PMC13193872; doi:10.3389/fonc.2026.1806162)
Supplement: Supplementary Table 1 — Patient characteristics of 77 patients with p16-negative HNSCC. [file DataSheet1.pdf]

Supplemental Table S1. Patient characteristics of 77 patients with p16-negative HNSCC. HNSCC: Head and Neck Squamous Cell Carcinoma; IQR: interquartile range; T stage: size of tumors staged 1-4; N stage: presence of lymph node metastases, staged 0-3; R stage: indicates rest of tumor in resected tumor margins, 0 – free of tumor, 1 – tumor in situ, x – cannot be clarified pathologically; UICC: Union for International Cancer Control; RTx: Radiation only; RCTx: Radiochemotherapy; CTx: Chemotherapy only.

Variable	Category	Patients n (%)	Median (Range; IQR)
Age			59.3 (30.0-78.1; 11.8)
	< 60	40 (51.9)	
	≥ 60	37 (48.1)	
Gender	Male	65 (84.4)	
	Female	12 (15.6)	
Tumor Site	Oral cavity	18 (23.4)	
	Oropharynx	16 (20.8)	
	Larynx	22 (28.6)	
	Hypopharynx	21 (27.3)	
T stage	T1	8 (10.4)	
	T2	27 (35.1)	
	T3	17 (22.1)	
	T4	25 (32.5)	
N stage	N0	19 (24.7)	
	N1	4 (5.2)	
	N2	54 (70.1)	
	N3	0 (0)	
Grading	1	3 (3.9)	
	2	33 (42.9)	
	3	41 (53.2)	
R stage	0	43 (55.8)	
	1	28 (36.4)	
	x	6 (7.8)	
UICC stage	I	3 (3.9)	
	II	3 (3.9)	
	III	4 (5.2)	
	IVa	67 (87.0)	
	IVb	0 (0)	
Surgery	Yes	74 (96.1)	
	No	3 (3.9)	
Adjuvant treatment	None	16 (20.8)	
	RTx	32 (41.6)	
	CRTx	29 (37.7)	
	CTx	0 (0)	

Supplemental Table S2. Tissue area analyzed and densities of TAMs and M2-like TAMs.

Data are given as median, minimum to maximum range and interquartile range (IQR).TAMs: Tumor-associated macrophages.

**Variable**	**Median (Range; IQR)**
Total Area [mm^2]	8.3 (1.1-26.6; 7.0)
Total Stroma Area [mm^2]	3.4 (0.4-13.9; 4.8)
Total Tumor Area [mm^2]	3.8 (0.04-23.7; 4.7)
Total Density $CD68^+$ [cells/mm^2]	150.9 (1.5-1018; 203.1)
Total Stroma Density $CD68^+$ [cells/mm^2]	104.6 (0.0-1119; 225.9)
Total Tumor Density $CD68^+$ [cells/mm^2]	113.5 (0.6-1352; 202.7)
Total Density CD68+$CD163^+$ [cells/mm^2]	18.2 (0.0-538.4; 41.6)
Total Stroma Density CD68+$CD163^+$ [cells/mm^2]	10.8 (0.0-960.5; 46.1)
Total Tumor Density CD68+ $CD163^+$ [cells/mm^2]	9.1 (0.0-695.8; 38.4)

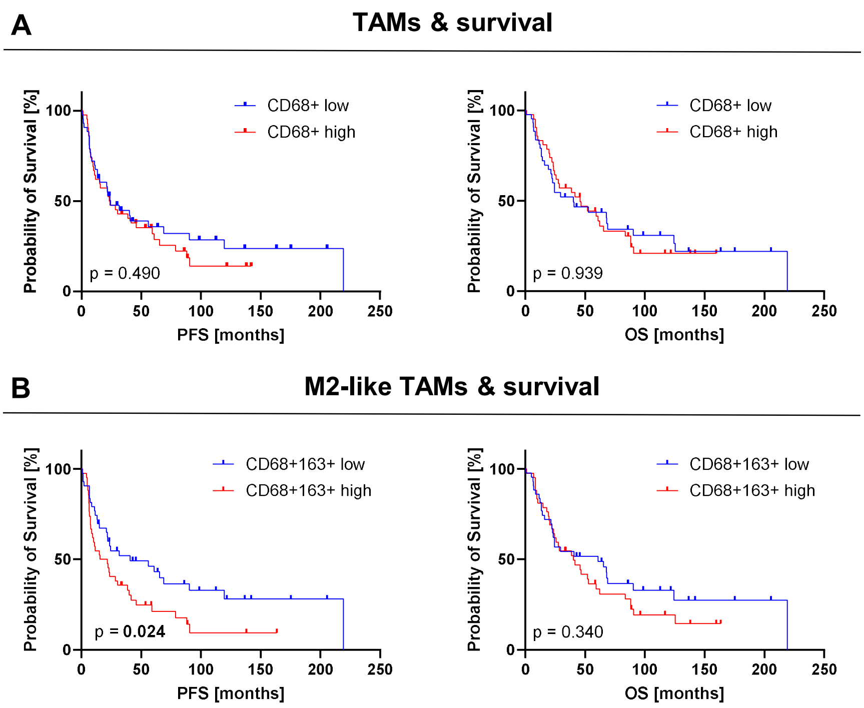

Supplemental Figure S1: Survival analysis in the total study cohort in relation to TAM- and M2-like TAM infiltrations. A Progression-free survival (PFS) and overall survival of the total study cohort (n = 85) in relation to their TAM (CD68+) densities (median split). **B** Progression-free survival (PFS) and overall survival of the total study cohort (n = 85) in relation to their M2-like TAM (CD68+CD163+) densities (median split). TAM: Tumor-associated macrophages; p: p-value. Bold: significant p-value < 0.05.

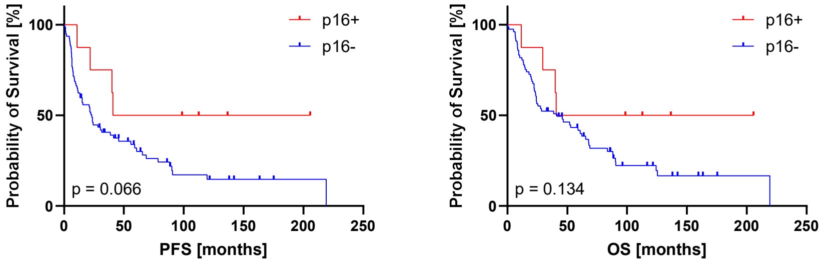

Supplemental Figure S2. Univariate survival analysis and p16-status. Progression-free (PFS) and overall survival (OS) depending on p16-status in HNSCCs (n = 85). p: p-value.

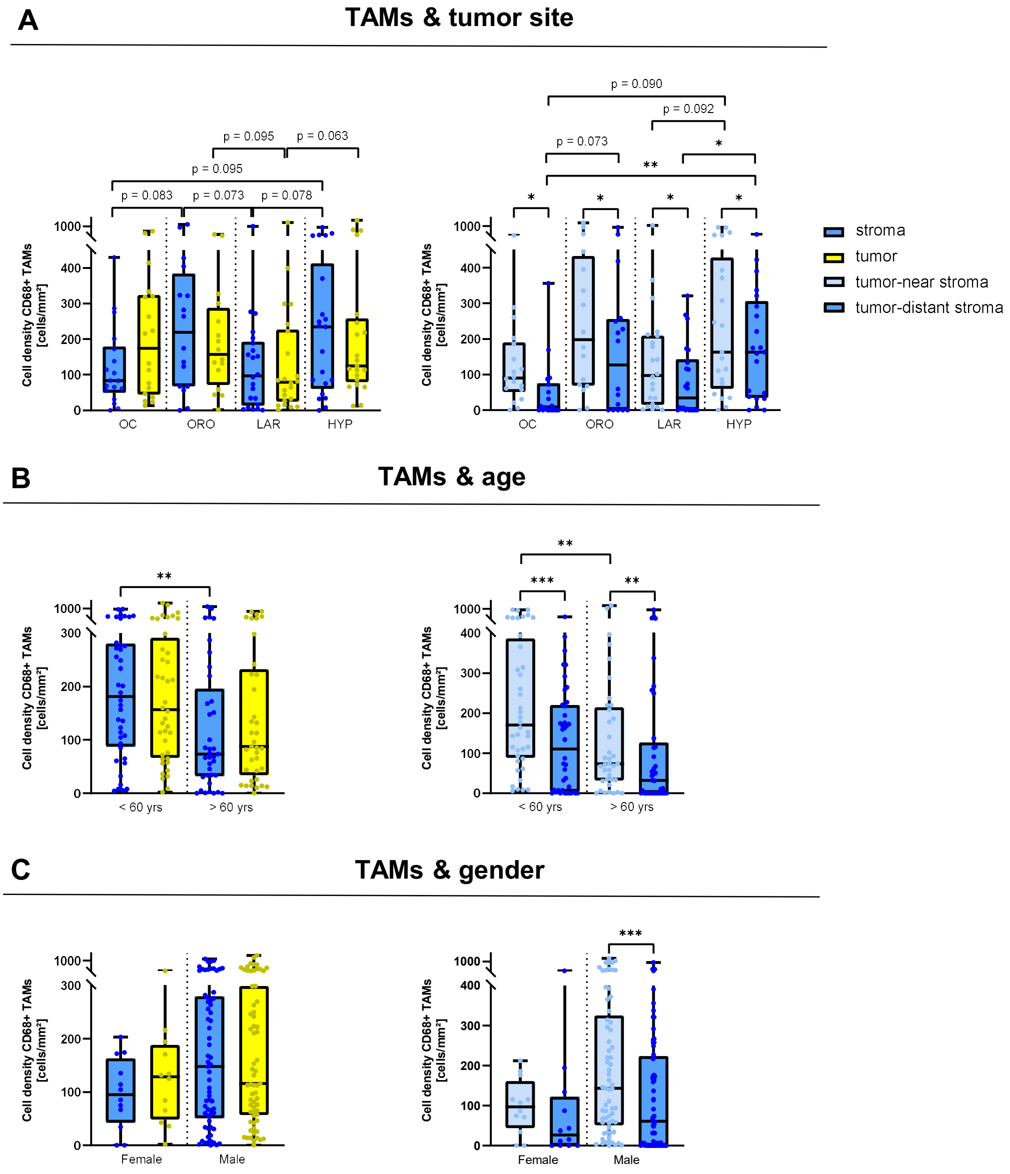

Supplemental Figure S3: Spatial distribution of TAMs related to clinicopathological features. Infiltration of CD68+ tumor-associated macrophages (TAMs) into tumor stroma (blue) and tumor cell nests (yellow) (left panel) and into tumor-near stroma (bright blue) and tumor-distant stroma (dark blue) (right panel) related to tumor site (A), age (B), and gender (C) in p16-negative head and neck squamous cell carcinomas (n = 77).* $p < 0.05$; ** $p < 0.01$.

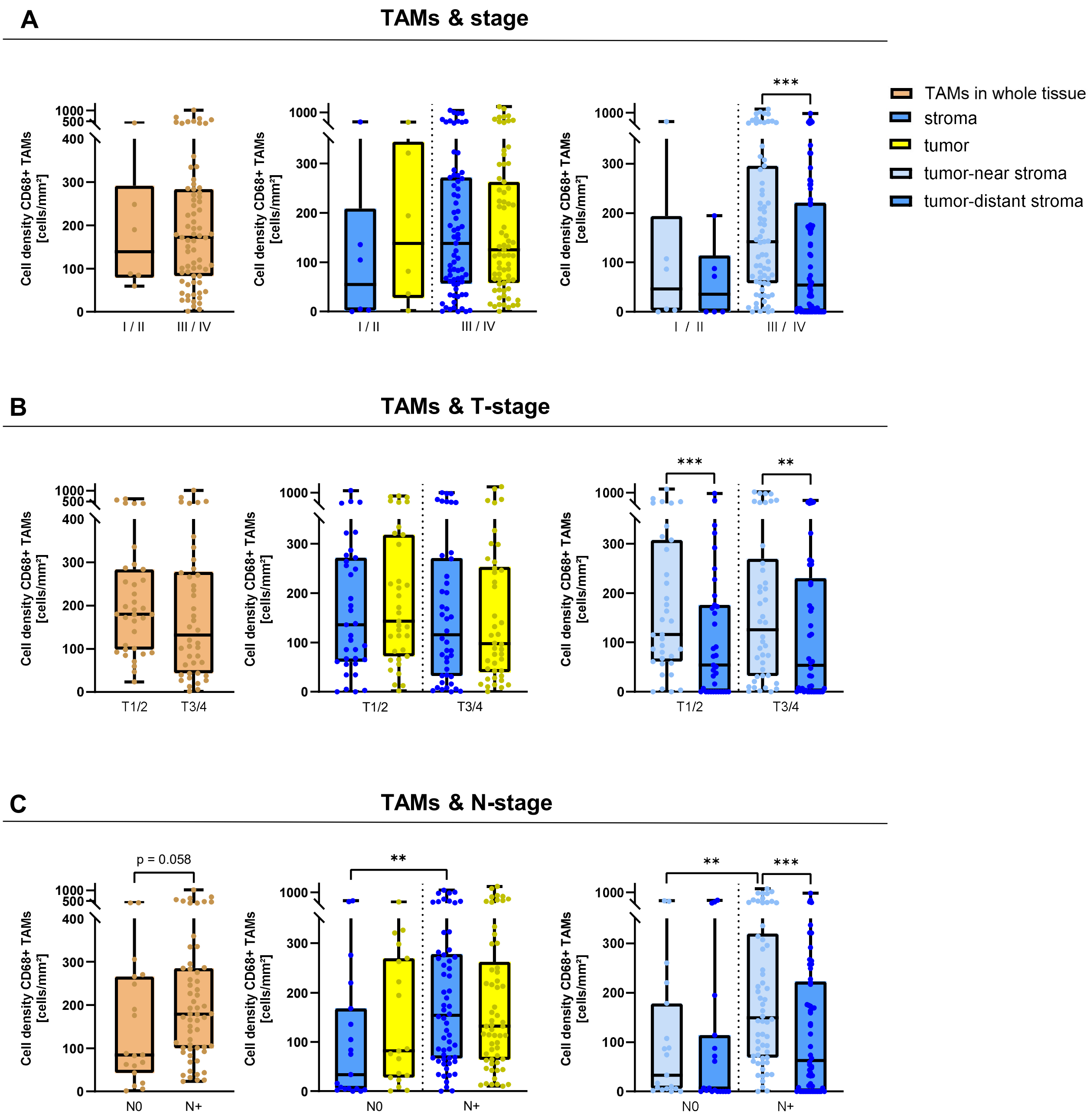

Supplemental Figure S4: Spatial distribution of TAMs related to clinicopathological features. Infiltration of CD68+ tumor-associated macrophages (TAMs) into whole tissue (orange, left panel), tumor stroma (blue) and tumor cell nests (yellow) (middle panel) and into tumor-near stroma (bright blue) and tumor-distant stroma (dark blue) (right panel) related to stage (A), T-stage (B), and N-stage (C) in p16-negative head and neck squamous cell carcinomas (n = 77).* $p < 0.05$; ** $p < 0.01$.

M2-like TAMs

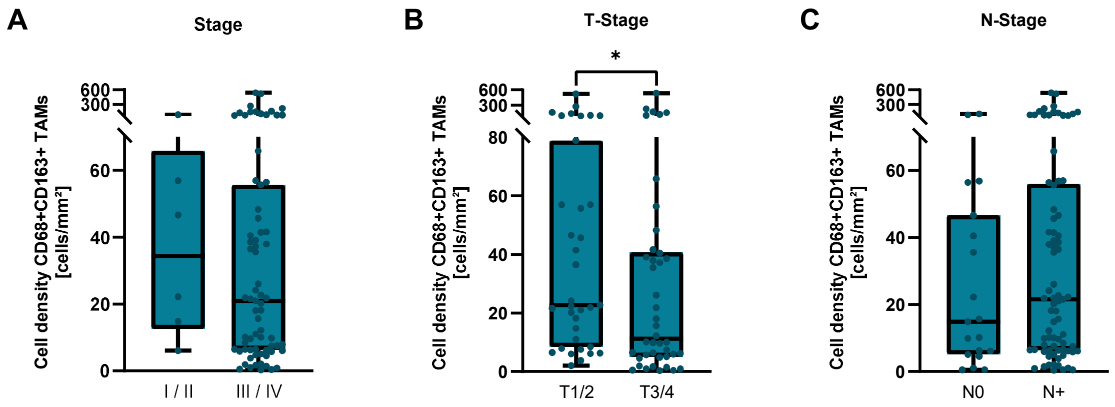

Supplemental Figure S5: CD68+CD163+ M2-like TAM infiltration of whole tumor tissue in p16-negative head and neck squamous cell carinomas (n = 77) in relation to stage (A), T-stage (B), and N-stage (C). * $p < 0.05$, ** $p < 0.01$. TAM: Tumor-associated macrophages.

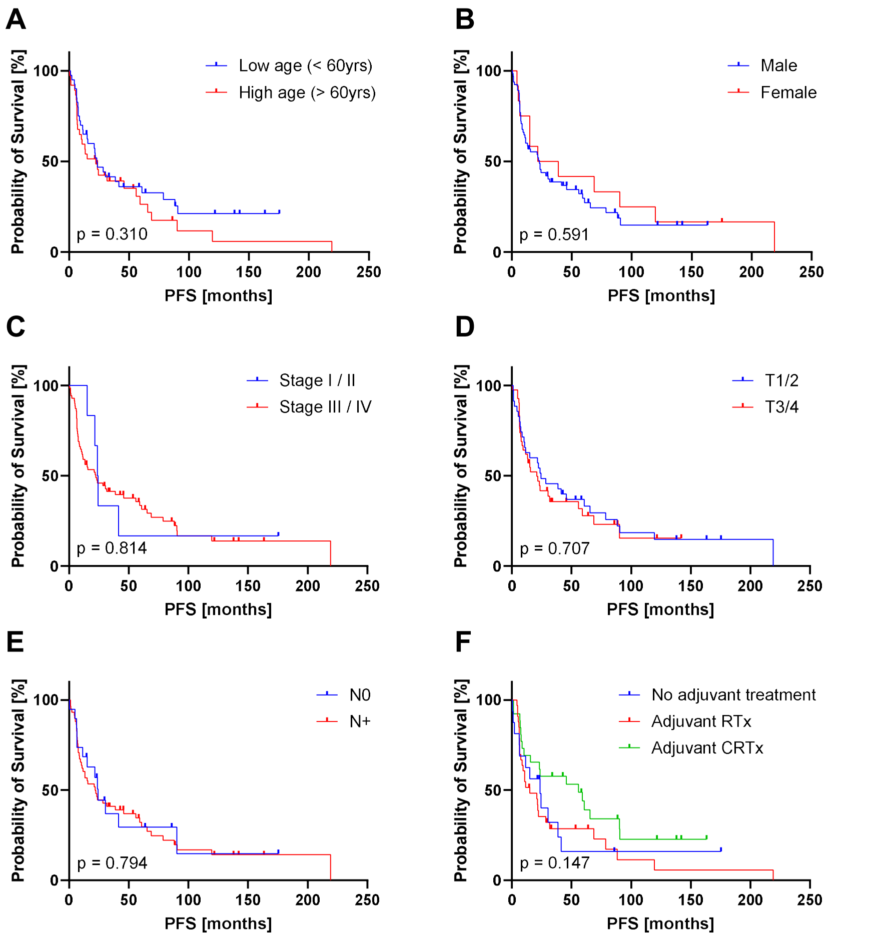

Supplemental Figure S6. Progression-free survival (PFS) analysis of patients with p-16 negative head and neck squamous cell carcinomas (HNSCCs) related to clinicopathological features. PFS analysis of p16-negative HNSCCs (n = 77) in relation to **A** age, **B** gender, **C** stage, **D** T-stage, **E** N-stage, and **F** adjuvant treatment. p: p-value.

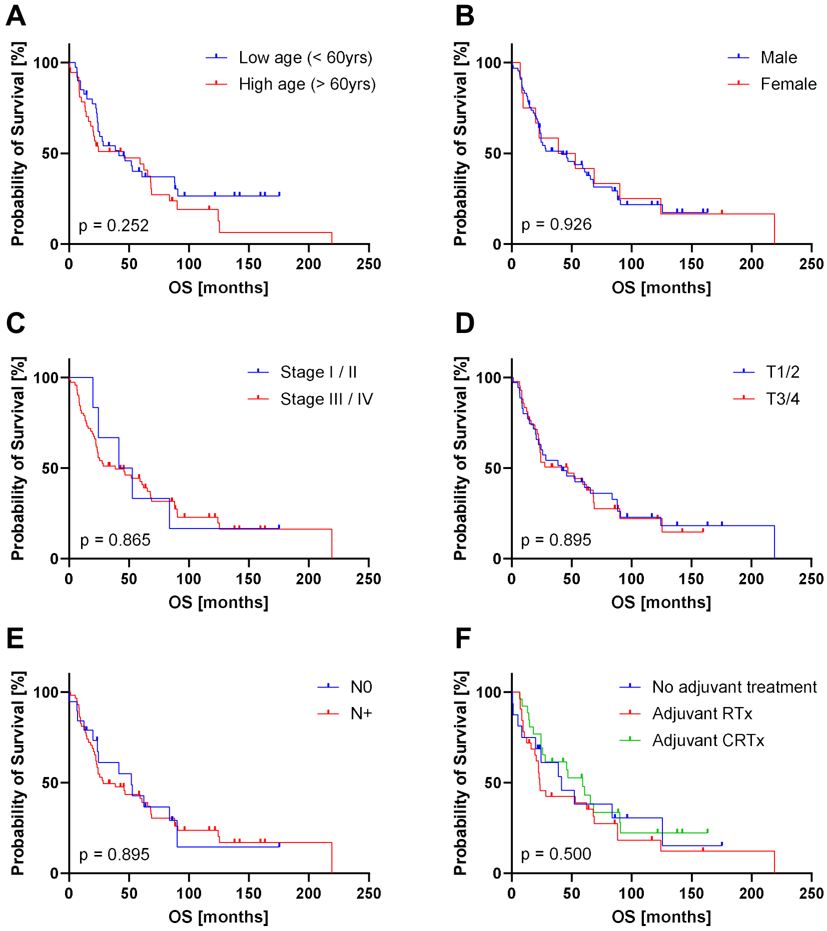

Supplemental Figure S7. Overall survival (OS) analysis of patients with p-16 negative head and neck squamous cell carcinomas (HNSCCs) related to clinicopathological features. OS analysis of p16-negative HNSCCs (n = 77) in relation to **A** age, **B** gender, **C** stage, **D** T-stage, **E** N-stage, and **F** adjuvant treatment. p: p-value.

A

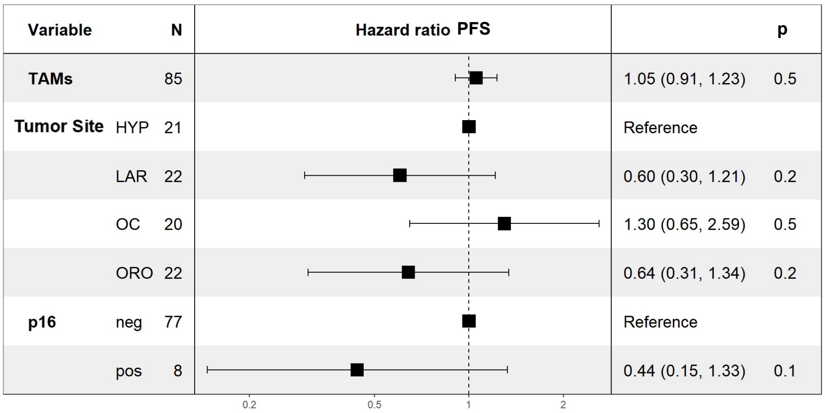

B

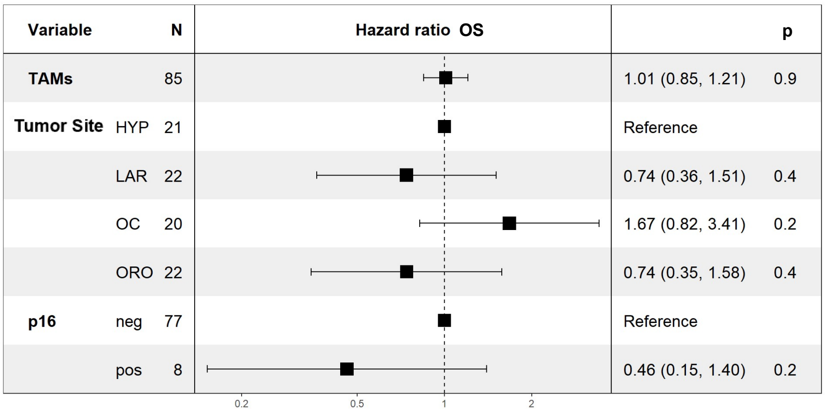

Supplemental Figure S8: Multivariate analysis of total TAM densities including the covariates tumor site, and p16-status. Multivariate analysis of **A** progression-free survival (PFS) and **B** overall survival (OS). N: Absolute number of included head and neck squamous cell cancers; p: p-value; TAMs: CD68+ Tumor-associated macrophages; HYP: Hypopharyngeal carcinoma; LAR: Laryngeal carcinoma; OC: Oral Cavity; ORO: Oropharyngeal carcinoma; neg: p16-negative; pos: p16-positive.
